# Supplementary material for: Chemical Fractionations of Lead and Zinc in the Contaminated Soil Amended with the Blended Biochar/Apatite
Source: Molecules. 2022 Nov 19;27(22):8044. doi: 10.3390/molecules27228044 (PMC9698809; doi:10.3390/molecules27228044)
Supplement: Supplementary file 1 [file molecules-27-08044-s001.zip › molecules-1981463-supplementary.pdf]

Supplementary Information

Table S1: Operating parameter of microwave digestion system for digesting soil samples

Nr	Operating parameters of microwave digestion Mars 6	
1	Power	(~ 1200 W)
2	Rising temperature duration	10 minutes
3	Holding time	15 minutes
4	Digestion temperature	170 °C
5	Cooling time	20 minutes

Table S2. Parameter of ICP-MS Agilent 7900 and recovery values of elements in sediment reference material MESS-4

Operating conditions of ICP-MS Agilent 7900			
High-Frequency Power (w)	~1600		
Sampling depth (mm)	~10		
Carrier gas flow rate (L/min)	~0,7		
Auxiliary gas flow rate (L/min)	~0,3		
Peristaltic nebulizer (concentric glass)	MicroMist		
Spray chamber temperature (°C)	2		
Helium gas flow rate (mL/min)	~4,3		
Hydrogen gas flow rate (mL/min)	~4,2		
Peristaltic pump speed	0,1 (0,5 mL/min)		
Internal standard	^{115}In		
Isotopes	^{208}Pb, ^{111}Cd, ^{66}Zn		
LOD and LOQ values of metals using ICP-MS	Element	LOD (ng/L)	LOQ (ng/L)
	Pb	13.7	41.5
	Cd	2.4	7.2
	Zn	1.5	4.5
Recovery values of metal elements (%) for MESS-4	Element	Recovery (%)	RSD (%)
	Pb	109.27	9.82
	Cd	92.11	5.88
	Zn	103.22	13.47

Table S3. Fractionation extraction procedure of Tessier

Step	Fractions	Reagent extractions/ conditions
1	Exchangeable (F1)	10 mL NH_4OAc 1M, 1 hour shaking
2	Carbonates bound (F2)	20 mL NH_4OAc 1M/HOAc pH = 5,<br>5 hours shaking
3	Fe and Mn oxides bound (F3)	20 mL $NH_2OH.HCl$ 0,04M/HOAc 25%,<br>5 hours shaking
4	Organic matter bound (F4)	10 mL NH_4OAc 3,2M + HNO_3 20%,<br>0.5 hour shaking
5	Residual (F5)	50 mL HNO_3 + HCl (v/v = 1:3),<br>0.5 hour shaking

Table S4. Fractionation of soil samples after 30 days of incubation with biochar and apatite

Metal	Sample	F1 (mg/kg)	F2 (mg/kg)	F3 (mg/kg)	F4 (mg/kg)	F5 (mg/kg)	Sum (mg/kg)
**Pb**	CS	495.77 ± 5.20	1893.7 ± 36.30	31.58 ± 0.5	18.39 ± 4.12	535.07 ± 28.66	3022.71
	PB4:3	455.73 ± 15.24	1935.3 ± 175.94	33.13 ± 4.25	21.01 ± 1.40	624.03 ± 39.59	3019.22
	PB4:5	328.46 ± 7.32	1920.3 ± 92.35	35.23 ± 7.62	32.04 ± 1.47	632.12 ± 32.91	2948.19
	PB4:10	275.15 ± 31.29	1880.7 ± 67.18	31.29 ± 0.28	49.89 ± 1.05	581.29 ± 21.36	2818.28
	PB4A3	324.88 ± 16.60	1995.0 ± 116.52	26.14 ± 5.10	9.89 ± 0.62	544.69 ± 5.91	2900.60
	PB4A5	275.37 ± 9.01	1917.7 ± 83.16	25.83 ± 2.55	9.57 ± 0.35	546.62 ± 25.65	2775.06
	PB6:3	319.82 ± 39.99	1842.3 ± 111.51	33.63 ± 14.77	27.62 ± 2.57	534.81 ± 74.03	2758.22
	PB6:5	295.26 ± 12.05	1925.3 ± 211.53	31.20 ± 7.08	22.45 ± 5.68	523.78 ± 51.93	2798.02
	PB6:10	234.55 ± 18.27	1910.7 ± 407.20	30.88 ± 0.93	21.80 ± 6.81	572.05 ± 32.94	2769.94
	PB6A3	256.83 ± 28.50	1847.7 ± 227.91	26.39 ± 1.38	58.88 ± 4.62	585.20 ± 0.28	2774.94
	PB6A5	252.83 ± 17.81	1819.0 ± 70.15	30.11 ± 2.70	76.96 ± 8.24	578.74 ± 22.03	2757.65
**Zn**	CS	424.82 ± 4.69	805.7 ± 44.07	64.83 ± 0.50	9.28 ± 1.21	699.18 ± 20.92	1995.92
	PB4:3	416.97 ± 6.93	778.0 ± 9.85	64.82 ± 4.25	20.00 ± 0.90	671.12 ± 8.71	1958.77
	PB4:5	348.97 ± 34.08	758.3 ± 16.77	68.11 ± 7.62	19.05 ± 2.40	622.42 ± 53.52	1816.88
	PB4:10	277.69 ± 16.52	735.3 ± 75.06	59.93 ± 0.28	58.00 ± 0.90	780.12 ± 46.03	1811.07
	PB4A3	326.84 ± 23.90	688.7 ± 25.81	59.44 ± 5.10	48.70 ± 0.40	684.16 ± 55.57	1807.82
	PB4A5	279.72 ± 32.17	794.0 ± 36.50	56.15 ± 2.55	51.55 ± 1.53	648.58 ± 10.37	1830.00
	PB6:3	388.75 ± 27.62	752.0 ± 27.87	74.95 ± 14.77	21.10 ± 3.27	575.86 ± 14.85	1812.67
	PB6:5	321.33 ± 22.01	707.7 ± 21.73	70.73 ± 7.08	19.54 ± 5.97	682.85 ± 16.40	1802.12
	PB6:10	302.89 ± 22.68	728.7 ± 30.75	63.24 ± 0.93	17.83 ± 0.86	745.06 ± 19.90	1857.68
	PB6A3	308.82 ± 20.76	727.0 ± 17.06	59.29 ± 1.38	15.63 ± 0.68	707.40 ± 3.65	1818.17
	PB6A5	311.78 ± 11.83	770.0 ± 48.51	57.67 ± 2.70	18.41 ± 0.74	685.24 ± 13.84	1843.09

Table S5. Proportion of chemical fractions of Pb, Zn in soils after 30 days of incubation with biochar and apatite ore (%).

Metal	Sample	F1 (%)	F2 (%)	F3 (%)	F4 (%)	F5 (%)
Pb	CS	16.67 ± 0.17^{a}	63.66 ± 1.22^{b}	1.06 ± 0.02^{b}	0.62 ± 0.14^{d}	17.99 ± 0.96^{c}
	PB4:3	14.85 ± 0.50^{b}	63.06 ± 5.73^{ab}	1.08 ± 0.14^{ab}	0.68 ± 0.05^{d}	20.33 ± 1.29^{ab}
	PB4:5	11.14 ± 0.25^{c}	65.14 ± 3.13^{ab}	1.19 ± 0.26^{ab}	1.09 ± 0.05^{c}	21.44 ± 1.12^{ab}
	PB4:10	9.76 ± 1.11^{e}	66.73 ± 2.38^{ab}	1.11 ± 0.01^{a}	1.77 ± 0.04^{b}	20.63 ± 0.76^{ab}
	PB4A3	11.20 ± 0.57^{c}	68.78 ± 4.02^{ab}	0.90 ± 0.18^{ab}	0.34 ± 0.02^{e}	18.78 ± 0.20^{bc}
	PB4A5	9.92 ± 0.32^{e}	69.10 ± 3.00^{a}	0.93 ± 0.09^{b}	0.34 ± 0.01^{e}	19.70 ± 0.92^{bc}
	PB6:3	11.60 ± 1.45^{c}	66.79 ± 4.04^{ab}	1.22 ± 0.54^{ab}	1.00 ± 0.09^{c}	19.39 ± 2.68^{abc}
	PB6:5	10.55 ± 0.43^{cd}	68.81 ± 7.56^{ab}	1.12 ± 0.25^{ab}	0.80 ± 0.20^{d}	18.72 ± 1.86^{abc}
	PB6:10	8.47 ± 0.66^{e}	68.98 ± 4.70^{ab}	1.11 ± 0.03^{a}	0.79 ± 0.25^{d}	20.65 ± 1.19^{ab}
	PB6A3	9.26 ± 1.03^{de}	66.58 ± 8.21^{ab}	0.95 ± 0.05b	2.12 ± 0.17^{a}	21.09 ± 0.01^{a}
	PB6A5	9.17 ± 0.65^{e}	65.96 ± 2.54^{ab}	1.09 ± 0.10^{ab}	2.79 ± 0.30^{a}	20.99 ± 0.80^{a}
Zn	CS	20.89 ± 0.23^{a}	40.37 ± 2.21^{a}	3.25 ± 0.03^{b}	0.46 ± 0.06^{e}	35.03 ± 1.05^{c}
	PB4:3	21.69 ± 0.56^{a}	39.72 ± 0.50^{ab}	3.31 ± 0.22^{ab}	1.02 ± 0.05^{c}	34.26 ± 0.44^{c}
	PB4:5	19.21 ± 1.88^{ab}	41.74 ± 0.92^{a}	3.75 ± 0.42^{a}	1.05 ± 0.13^{c}	34.26 ± 2.95^{cd}
	PB4:10	14.53 ± 0.86^{c}	38.48 ± 3.93^{ab}	3.14 ± 0.01^{c}	3.03 ± 0.05^{a}	40.82 ± 2.41^{a}
	PB4A3	18.08 ± 1.32^{b}	38.10 ± 1.43^{ab}	3.29 ± 0.28^{abc}	2.69 ± 0.02^{b}	37.84 ± 3.07^{abc}
	PB4A5	15.29 ± 1.76	43.39 ± 1.99^{a}	3.07 ± 0.14^{bc}	2.82 ± 0.08^{a}	35.44 ± 0.57^{c}
	PB6:3	21.45 ± 1.52^{ab}	41.49 ± 1.54^{a}	4.13 ± 0.81^{a}	1.16 ± 0.18^{c}	31.77 ± 0.82^{d}
	PB6:5	17.83 ± 1.22^{b}	39.27 ± 1.21^{ab}	3.92 ± 0.39^{a}	1.08 ± 0.33^{c}	37.89 ± 0.91^{ab}
	PB6:10	16.30 ± 1.22^{bc}	39.22 ± 1.66^{ab}	3.40 ± 0.05^{a}	0.96 ± 0.05^{c}	40.11 ± 1.07^{a}
	PB6A3	16.99 ± 1.14^{bc}	39.99 ± 0.94^{ab}	3.26 ± 0.08^{ab}	0.86 ± 0.04^{d}	38.91 ± 0.20^{ab}
	PB6A5	16.92 ± 0.64^{bc}	41.78 ± 2.63^{a}	3.13 ± 0.15^{bc}	1.00 ± 0.04^{c}	37.18 ± 0.75^{abc}

Note: Mean ± SD, n =3; Means followed by the same letters (a – h) within the same column are not significantly different at a 5% level probability.
